# Crystal structure of 3-ferrocenyl-1-phenyl-1*H*-pyrrole, [Fe(η^5^-C_5_H_4_^*c*^C_4_H_3_ *N*Ph)(η^5^-C_5_H_5_)]

**DOI:** 10.1107/S2056989015024214

**Published:** 2016-01-01

**Authors:** Ulrike Pfaff, Marcus Korb, Heinrich Lang

**Affiliations:** a Technische Universität Chemnitz, Fakultät für Naturwissenschaften, Institut für Chemie, Anorganische Chemie, D-09107 Chemnitz, Germany

**Keywords:** crystal structure, ferrocene, pyrrole, Negishi *C*,*C* cross-coupling

## Abstract

The mol­ecular structure of the title compound, [Fe(C_5_H_5_)(C_15_H_12_N)], consists of a ferrocene moiety with an *N*-phenyl­pyrrole heterocycle bound to one cyclo­penta­dienyl ring. The 1,3-disubstitution of the pyrrole results in an L-shaped arrangement of the mol­ecule with plane inter­sections of 2.78 (17)° between the pyrrole and the *N*-bonded phenyl ring and of 8.17 (18)° between the pyrrole and the cyclo­penta­dienyl ring. In the crystal, no remarkable inter­molecular inter­actions are observed

## Abstract 2

The mol­ecular structure of 3-ferrocenyl-*N*-phenyl­pyrrole, [Fe(η^5^-C_5_H_4_^*c*^C_4_H_3_ *N*Ph)(η^5^-C_5_H_5_)] has an L-type shape, with the *N*-phenyl­pyrrole moiety fused with the cycla­penta­dienyl ring being approximately coplanar.

## Chemical context   

Ferrocenyl-substituted pyrroles have been investigated in electron-transfer studies (for example, see: Hildebrandt *et al.*, 2011*a* ▸,*b* ▸; Hildebrandt & Lang, 2011 ▸, 2013 ▸; Pfaff *et al.*, 2013 ▸, 2015*a* ▸; Korb *et al.*, 2014 ▸; Yu-Qiang *et al.*, 2015 ▸), demonstrating that pyrroles are well suited to examine intra­molecular metal–metal inter­actions in mixed-valent species, when compared to other heterocycles such as furan, thio­phene, phosphole or siloles (Hildebrandt *et al.*, 2013 ▸, 2011 ▸; Pfaff *et al.*, 2015*a* ▸,*b* ▸; Lehrich *et al.*, 2014 ▸; Miesel *et al.*, 2013 ▸, 2015 ▸; Speck *et al.*, 2012*a* ▸, 2014 ▸, 2015 ▸). As has been shown in the study of 3,4-diferrocenyl pyrroles [3,4-*Fc*_2_-^c^C_4_H_2_N*R*; *Fc* = Fe(η^5^-C_5_H_4_)(η^5^-C_5_H_5_); *R* = Ph, SO_2_-4-MeC_6_H_4_, Si^*i*^Pr_3_; Korb *et al.*, 2014 ▸; Goetsch *et al.*, 2014 ▸], the compounds showed a low degree of delocalization between the formal *C*,*C* double and *C*,*C* single bonds, in contrast to 2,5-substituted pyrroles (Korb *et al.*, 2014 ▸). In addition, these compounds exhibit rather weak, broad inter-valence charge-transfer transitions in spectro-electrochemical investigations in the NIR region of the mixed-valent species. Lower redox splittings were also detected for such compounds. These results indicate that in mono-oxidized 3,4-diferrocenyl-substituted pyrroles the intra­molecular electron transfer is quite weak. In a continuation of this work, we present herein the synthesis and crystal structure of 3-ferrocenyl-*N*-phenyl­pyrrole, (I), [Fe(η^5^-C_5_H_4_^*c*^C_4_H_3_ *N*Ph)(η^5^-C_5_H_5_)]. The synthesis of this compound was realized using typical Negishi *C*,*C* cross-coupling reaction conditions.
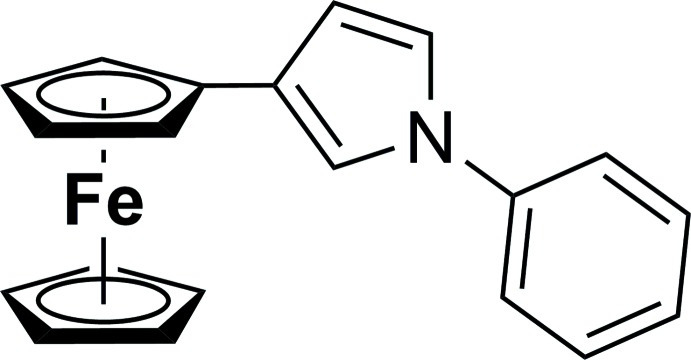


## Structural commentary   

**Figure 1:**
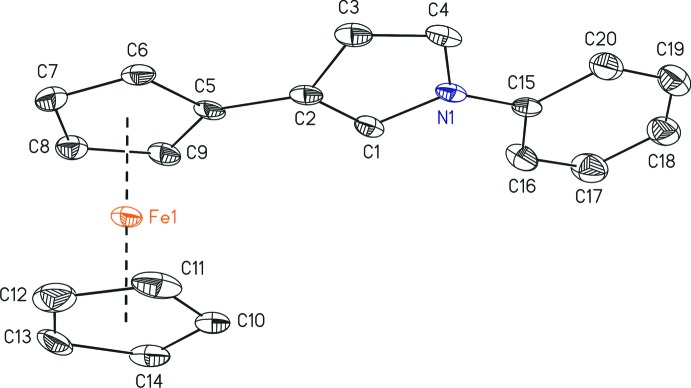
The mol­ecular structure of (I), with displacement ellipsoids drawn at the 50% probability level. All H atoms have been omitted for clarity.

The 1,3-disubstitution of the pyrrole ring in compound (I) results in an L-type shape of the mol­ecule with a bending of 34.882 (2)° of the three catenated ring systems, as calculated by the angle between the centroids of the respective cyclo­penta­dienyl, pyrrole and phenyl rings. The three rings are nearly coplanar, with plane inter­sections of 8.17 (18)° between the central pyrrole ring with the cyclo­penta­dienyl ring and of 2.78 (17)° between the pyrrole ring and the *N*-bound phenyl ring (Fig. 1 ▸). The ferrocenyl substituent itself exhibits a nearly eclipsed conformation with a torsion angle of −12.2 (2)°. The 3-substitution affects the lengths of the C=C bonds in the pyrrole ring, resulting in a shortening to 1.349 (4) Å of the H3C3=C4H4 bond compared to 1.378 (4) Å for the C2=C1H1 bond. However, the unsymmetrical substitution pattern does not significantly affect the C—N bonds of the pyrrole ring system.

## Supra­molecular features   

**Figure 2:**
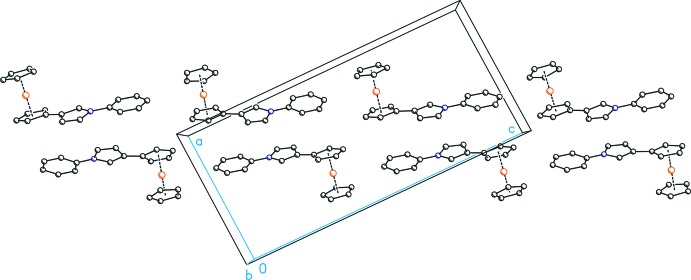
Packing of the mol­ecules in the crystal structure of (I) in a view along [010]. All H atoms have been omitted for clarity.

**Figure 3:**
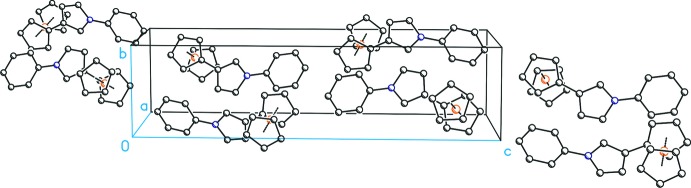
Packing of the mol­ecules in the crystal structure of (I) resulting in a wave-type arrangement along [001]. All H atoms have been omitted for clarity.

In the crystal packing of (I), the *N*-phenyl­pyrrole moieties are directed along [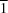
01] with alternating directions for adjacent rows (Fig. 2 ▸). The bent shape caused by the 3-substitution pattern furthermore results in a corrugated arrangement of the mol­ecules along [001] (Fig. 3 ▸). Inter­estingly, no remarkable intra- or inter­molecular inter­actions, *e.g*. in the form of π–π inter­actions, are observed. Therefore it appears that the crystal packing is mainly dominated by van der Waals forces.

## Database survey   

A CSD database search (Groom & Allen, 2014 ▸) for 3-ferrocenyl five-membered aromatics gave eleven results with seven of them disubstituted in the 3- and 4-positions including thio­phenes, like the super-crowded 3,3′,4,4′,5,5′-hexa­ferrocenyl-2,2′-bi­thio­phene (Speck *et al.*, 2012*b* ▸), 2,3,4,5-tetra­kis­(ferrocen­yl)thio­phene (Hildebrandt *et al.*, 2010 ▸) and also 1,1′-disubstituted ferrocenes bearing a 3-thienyl and a 3,5-bis­(tri­fluoro­meth­yl)phenyl substituent (Poppitz *et al.*, 2014 ▸). 1,3-Disubstituted thio­phenes are also reported (Speck *et al.*, 2012*a* ▸) due to the easy accessibility of each position. However, the 3- (and 4-) substitution of pyrroles is rather difficult, requiring sterically demanding *N*-substituents to block the 2- and 5-positions, *e.g. N*-triiso­propyl­silyl (Korb *et al.*, 2014 ▸; Goetsch *et al.*, 2014 ▸) or deactivating *p*-toluene­sulfonyl substituents (Korb *et al.*, 2014 ▸). Thus, several multiple ferrocenyl structures are known, including the super-crowded 2,3,4,5-tetra­ferrocenyl pyrrole bearing either an *N*-Me (Hildebrandt *et al.*, 2011*a* ▸) or *N*-Ph substituent (Hildebrandt *et al.*, 2011*b* ▸).

However, a single substituted pyrrole bearing just one ferrocenyl substituent in the 3-position has not been reported so far. It should be noted that related structures like 3-ferrocenyl male­imides (Mathur *et al.*, 2012 ▸) and a 3-ferrocenyl boron-dipyrromethene (Dhokale *et al.*, 2013 ▸) are reported bearing one ferrocenyl substituent.

Comparing the plane inter­sections between the ferrocenyl and the pyrrolic ring systems, compound (I) exhibits the most coplanar torsion of 8.17 (18)° followed by 3,4-diferrocenyl-*N*-tosyl pyrrole (Korb *et al.*, 2014 ▸) with 19.855 (6)° or, in the case of male­imides, the 3-bromo-4-ferrocenyl-*N*-phenyl-derivative with 9.8° (Hildebrandt *et al.*, 2012 ▸).

The smallest inter­section between the phenyl and pyrrole rings are reported with 5.4° for a 3-ferrocenyl-pyrrolo­[1,2-*a*]quinoxaline (Guillon *et al.*, 2011 ▸), due to the hindered rotation of the *N*—C_Ph_ bond. However, comparable derivatives with free rotable *N*-aromatics exhibit torsions above 35° (Hilde­brandt *et al.*, 2012 ▸).

## Synthesis and crystallization   

3-Bromo-*N*-phenyl­pyrrole was prepared from 2-bromo-*N*-phenyl­pyrrole according to the synthetic methodology reported by Choi *et al.* (1998 ▸). The synthesis of ferrocenyl pyrrole (I) was realized using typical Negishi *C*,*C* cross-coupling reaction conditions by reacting ferrocenyl zinc chloride with 3-bromo-*N*-phenyl­pyrrole (Negishi *et al.*, 1977 ▸).

**Synthesis of (I):** Ferrocene (0.35 g, 1.88 mmol) and 0.125 eq of KO^*t*^Bu (0.03 g, 0.23 mmol) were dissolved in 20 ml of tetra­hydro­furan and the respective solution was cooled to 193 K. Afterwards, 2 eq of ^*t*^butyl­lithium (2.4 ml, 3.76 mmol, 1.6 *M* in ^*n*^penta­ne) were added dropwise *via* a syringe and the reaction solution was stirred for 1 h. Then, 1 eq of [ZnCl_2_·2thf] (0.53 g, 1.88 mmol) was added in a single portion. The reaction mixture was stirred for additional 30 min at 273 K. Afterwards, 0.25 mol-% of [Pd(CH_2_C(CH_3_)_2_P(^*t*^C_4_H_9_)_2_)(*μ*-Cl)]_2_ (3.2 mg, 0.47 mmol) and 3-bromo-*N*-phenyl­pyrrole (0.27 g, 1.24 mmol) were added in a single portion and stirring was continued overnight at 333–343 K. After evaporation of all volatiles, the crude product was worked-up by column chroma­tography (silica, column size: 1.5 x 10 cm) using an *n*-hexa­ne/diethyl ether mixture (ratio 10:1; *v*/*v*) as the eluent. The first fraction contained ferrocene, while thereafter compound (I) was eluted as an orange phase. Single crystals of (I), suitable for single crystal diffraction analysis, were obtained by slow evaporation of a saturated di­chloro­methane/methanol (ratio 1:1 *v*/*v*) solution containing (I) at ambient temperature. Yield: 0.16 g (0.48 mmol, 39% based on 3-bromo-*N*-phenyl­pyrrole). IR data [KBr, cm^−1^] ν: 749 (*s*, δ _o.o.p.=C—H_), 1512 (*s*, ν_C=C_), 1599 (*m*, ν_C=C_), 3055, 3084 (*w*, ν_=C—H_). ^1^H NMR (CDCl_3_, p.p.m.) δ: 4.08 (*s*, 5 H, C_5_H_5_), 4.21 (*pt*, ^3+4^ *J*_H,H_ = 1.90 Hz, 2 H, C_5_H_4_), 4.48 (*pt*, ^3+4^ *J*_H,H_ = 1.90 Hz, 2 H, C_5_H_4_), 6.44 (*dd*, ^3^ *J*_H4,H5_ = 2.9 Hz, ^4^ *J*_H4,H2_ = 1.7 Hz, 1 H, H-4), 7.05 (*dd*, ^3^ *J*_H5,H4_ = 2.8 Hz, ^4^ *J*_H5,H2_ = 2.3 Hz, 1 H, H-5), 7.12 (*dd*, ^4^ *J*_H2,H5_ = 2.3 Hz, ^4^ *J*_H2,H4_ = 1.7 Hz, 1 H, H-2), 7.22–7.25 (*m*, 1 H, C_6_H_5_/*p*-H), 7.40–7.45 (*m*, 4 H, C_6_H_5_). ^13^C{^1^H} NMR (CDCl_3_, p.p.m.) δ: 66.19 (C_5_H_4_), 67.86 (C_5_H_4_), 69.60 (C_5_H_5_), 81.82 (C_*i*_-C_5_H_4_), 109.97 (C-4), 115.32 (C-2), 119.58 (C-5), 120.10 (C_6_H_5_), 124.07 (C_*i*_-C-3), 125.50 (C_6_H_5_), 129.70 (C_6_H_5_), 140.70 (C_*i*_-C_6_H_5_). HR–ESI–MS (*m*/*z*): calculated for C_20_H_17_NFe: 327.0705, found: 327.0715 (*M*)^+^. Analysis calculated for C_20_H_17_NFe (327.20 g/mol) (%): C, 73.41; H, 5.24; N, 4.28; found: C, 72.99; H, 5.31; N, 4.10. Mp.: 401 K. CV (mV): *E°′* = −123, Δ*E*_p_ = 74 (potentials *vs* FcH/FcH^+^).

## Refinement   

Crystal data, data collection and structure refinement details are summarized in Table 1 ▸. C-bonded aromatic hydrogen atoms were placed in calculated positions and constrained to ride on their parent atoms with *U*_iso_(H) = 1.2*U*_eq_(C) and a C—H distance of 0.93 Å.

## Supplementary Material

Crystal structure: contains datablock(s) I. DOI: 10.1107/S2056989015024214/wm5252sup1.cif

Structure factors: contains datablock(s) I. DOI: 10.1107/S2056989015024214/wm5252Isup2.hkl

CCDC reference: 1442943

Additional supporting information:  crystallographic information; 3D view; checkCIF report

## Figures and Tables

**Table 1:** Experimental details

Crystal data	
Chemical formula	[Fe(C_5_H_5_)(C_15_H_12_N)]
*M*_r_	327.19
Crystal system, space group	Monoclinic, *P*2_1_/*n*
Temperature (K)	110
*a*, *b*, *c* (Å)	10.9173 (8), 5.8011 (6), 23.085 (2)
β (°)	93.160 (7)
*V* (Å^3^)	1459.8 (2)
*Z*	4
Radiation type	Mo *K*α
μ (mm^−1^)	1.03
Crystal size (mm)	0.2 × 0.1 × 0.1

Data collection	
Diffractometer	Oxford Gemini S
Absorption correction	Multi-scan (*CrysAlis RED*; Oxford Diffraction, 2006 ▸)
*T*_min_, *T*_max_	0.192, 1.000
No. of measured, independent and observed [*I* > 2σ(*I*)] reflections	6054, 2858, 2198
*R*_int_	0.047
(sin θ/λ)_max_ (Å^−1^)	0.617

Refinement	
*R*[*F* ^2^ > 2σ(*F* ^2^)], *wR*(*F* ^2^), *S*	0.045, 0.110, 1.02
No. of reflections	2858
No. of parameters	199
H-atom treatment	H-atom parameters constrained
Δρ_max_, Δρ_min_ (e Å^−3^)	0.50, −0.75

Computer programs: *CrysAlis CCD* and *CrysAlis RED* (Oxford Diffraction, 2006 ▸), *SHELXS97* and *SHELXTL* (Sheldrick, 2008 ▸), *SHELXL2014* (Sheldrick, 2015 ▸), *ORTEP-3 for Windows* and *WinGX* (Farrugia, 2012 ▸) and *publCIF* (Westrip, 2010 ▸).

## supplementary crystallographic information

### Crystal data

**Table:**

[Fe(C_5_H_5_)(C_15_H_12_N)]	*F*(000) = 680
*M**_r_* = 327.19	*D*_x_ = 1.489 Mg m^−^^3^
Monoclinic, *P*2_1_/*n*	Mo *K*α radiation, λ = 0.71073 Å
*a* = 10.9173 (8) Å	Cell parameters from 1838 reflections
*b* = 5.8011 (6) Å	θ = 4.0–28.3°
*c* = 23.085 (2) Å	µ = 1.03 mm^−^^1^
β = 93.160 (7)°	*T* = 110 K
*V* = 1459.8 (2) Å^3^	Plate, orange
*Z* = 4	0.2 × 0.1 × 0.1 mm

### Data collection

**Table:**

Oxford Gemini S diffractometer	2198 reflections with *I* > 2σ(*I*)
Radiation source: fine-focus sealed tube	*R*_int_ = 0.047
Graphite monochromator	θ_max_ = 26.0°, θ_min_ = 3.2°
/w scans	*h* = −13→10
Absorption correction: multi-scan (*CrysAlis RED*; Oxford Diffraction, 2006)	*k* = −6→7
*T*_min_ = 0.192, *T*_max_ = 1.000	*l* = −28→25
6054 measured reflections	2 standard reflections every 50 reflections
2858 independent reflections	intensity decay: none

### Refinement

**Table:**

Refinement on *F*^2^	0 restraints
Least-squares matrix: full	Hydrogen site location: inferred from neighbouring sites
*R*[*F*^2^ > 2σ(*F*^2^)] = 0.045	H-atom parameters constrained
*wR*(*F*^2^) = 0.110	*w* = 1/[σ^2^(*F*_o_^2^) + (0.0503*P*)^2^] where *P* = (*F*_o_^2^ + 2*F*_c_^2^)/3
*S* = 1.02	(Δ/σ)_max_ = 0.001
2858 reflections	Δρ_max_ = 0.50 e Å^−^^3^
199 parameters	Δρ_min_ = −0.75 e Å^−^^3^

### Special details

**Table:**

Geometry. All e.s.d.'s (except the e.s.d. in the dihedral angle between two l.s. planes) are estimated using the full covariance matrix. The cell e.s.d.'s are taken into account individually in the estimation of e.s.d.'s in distances, angles and torsion angles; correlations between e.s.d.'s in cell parameters are only used when they are defined by crystal symmetry. An approximate (isotropic) treatment of cell e.s.d.'s is used for estimating e.s.d.'s involving l.s. planes.
Refinement. Refinement of *F*^2^ against ALL reflections. The weighted *R*-factor *wR* and goodness of fit *S* are based on *F*^2^, conventional *R*-factors *R* are based on *F*, with *F* set to zero for negative *F*^2^. The threshold expression of *F*^2^ > σ(*F*^2^) is used only for calculating *R*-factors(gt) *etc*. and is not relevant to the choice of reflections for refinement. *R*-factors based on *F*^2^ are statistically about twice as large as those based on *F*, and *R*- factors based on ALL data will be even larger.

### Fractional atomic coordinates and isotropic or equivalent isotropic displacement parameters (Å^2^)

**Table:**

	*x*	*y*	*z*	*U*_iso_*/*U*_eq_	
C1	0.5499 (3)	0.1196 (5)	0.24155 (12)	0.0173 (6)	
H1	0.5176	0.2615	0.2298	0.021*	
C2	0.5435 (3)	0.0236 (5)	0.29591 (12)	0.0165 (6)	
C3	0.6066 (3)	−0.1911 (5)	0.29421 (13)	0.0201 (7)	
H3	0.6183	−0.2940	0.3249	0.024*	
C4	0.6466 (3)	−0.2195 (5)	0.24043 (12)	0.0195 (7)	
H4	0.6901	−0.3461	0.2278	0.023*	
C5	0.4917 (3)	0.1248 (5)	0.34651 (13)	0.0165 (6)	
C6	0.4859 (3)	0.0136 (5)	0.40182 (12)	0.0186 (7)	
H6	0.5099	−0.1371	0.4103	0.022*	
C7	0.4375 (3)	0.1710 (5)	0.44135 (13)	0.0217 (7)	
H7	0.4251	0.1424	0.4802	0.026*	
C8	0.4112 (3)	0.3799 (5)	0.41138 (13)	0.0203 (7)	
H8	0.3783	0.5122	0.4272	0.024*	
C9	0.4436 (3)	0.3527 (5)	0.35336 (13)	0.0195 (7)	
H9	0.4352	0.4640	0.3244	0.023*	
C10	0.2029 (3)	−0.0235 (6)	0.30801 (14)	0.0249 (7)	
H10	0.2257	−0.0582	0.2708	0.030*	
C11	0.2121 (3)	−0.1733 (5)	0.35651 (15)	0.0281 (8)	
H11	0.2417	−0.3236	0.3566	0.034*	
C12	0.1686 (3)	−0.0549 (6)	0.40452 (15)	0.0302 (8)	
H12	0.1649	−0.1128	0.4419	0.036*	
C13	0.1314 (3)	0.1681 (5)	0.38567 (14)	0.0258 (8)	
H13	0.0985	0.2822	0.4085	0.031*	
C14	0.1533 (3)	0.1862 (5)	0.32592 (13)	0.0241 (7)	
H14	0.1375	0.3148	0.3026	0.029*	
C15	0.6417 (3)	0.0084 (5)	0.14879 (13)	0.0183 (7)	
C16	0.6046 (3)	0.2088 (5)	0.12047 (13)	0.0259 (7)	
H16	0.5595	0.3184	0.1396	0.031*	
C17	0.6347 (3)	0.2466 (6)	0.06357 (14)	0.0332 (8)	
H17	0.6088	0.3811	0.0447	0.040*	
C18	0.7025 (3)	0.0869 (6)	0.03478 (15)	0.0315 (8)	
H18	0.7229	0.1129	−0.0033	0.038*	
C19	0.7393 (3)	−0.1117 (6)	0.06342 (14)	0.0308 (8)	
H19	0.7850	−0.2204	0.0443	0.037*	
C20	0.7101 (3)	−0.1529 (5)	0.11972 (13)	0.0254 (8)	
H20	0.7359	−0.2881	0.1383	0.030*	
N1	0.6125 (2)	−0.0310 (4)	0.20734 (10)	0.0172 (5)	
Fe1	0.31350 (4)	0.11585 (7)	0.37338 (2)	0.01626 (16)	

### Atomic displacement parameters (Å^2^)

**Table:**

	*U*^11^	*U*^22^	*U*^33^	*U*^12^	*U*^13^	*U*^23^
C1	0.0098 (15)	0.0181 (14)	0.0240 (17)	0.0015 (13)	0.0006 (12)	−0.0006 (12)
C2	0.0070 (14)	0.0192 (15)	0.0232 (16)	−0.0028 (12)	−0.0017 (12)	0.0033 (12)
C3	0.0102 (15)	0.0227 (15)	0.0271 (17)	−0.0025 (13)	−0.0020 (13)	0.0043 (13)
C4	0.0098 (15)	0.0177 (14)	0.0306 (17)	0.0029 (13)	−0.0015 (13)	0.0014 (13)
C5	0.0051 (14)	0.0218 (15)	0.0227 (16)	−0.0020 (12)	0.0006 (12)	0.0030 (13)
C6	0.0096 (15)	0.0242 (16)	0.0216 (16)	−0.0006 (13)	−0.0035 (12)	0.0042 (13)
C7	0.0140 (16)	0.0309 (17)	0.0196 (16)	−0.0016 (14)	−0.0042 (13)	−0.0010 (13)
C8	0.0142 (16)	0.0216 (15)	0.0246 (16)	−0.0006 (13)	−0.0026 (13)	−0.0019 (13)
C9	0.0098 (15)	0.0222 (16)	0.0267 (17)	−0.0023 (13)	0.0016 (13)	0.0042 (13)
C10	0.0094 (16)	0.0397 (19)	0.0251 (17)	−0.0013 (15)	−0.0034 (13)	−0.0053 (15)
C11	0.0120 (16)	0.0218 (16)	0.050 (2)	−0.0024 (14)	−0.0049 (16)	−0.0019 (15)
C12	0.0154 (17)	0.045 (2)	0.0299 (19)	−0.0096 (16)	−0.0031 (14)	0.0086 (16)
C13	0.0083 (15)	0.0350 (19)	0.0342 (19)	−0.0024 (14)	0.0040 (14)	−0.0093 (15)
C14	0.0115 (16)	0.0283 (16)	0.0319 (18)	−0.0019 (14)	−0.0049 (14)	0.0060 (14)
C15	0.0052 (14)	0.0260 (16)	0.0236 (16)	−0.0037 (13)	−0.0008 (12)	−0.0020 (13)
C16	0.0182 (17)	0.0286 (17)	0.0312 (18)	0.0062 (15)	0.0040 (14)	0.0016 (15)
C17	0.027 (2)	0.041 (2)	0.0320 (19)	0.0071 (17)	0.0014 (16)	0.0096 (17)
C18	0.0201 (18)	0.049 (2)	0.0258 (18)	0.0030 (17)	0.0049 (15)	0.0022 (16)
C19	0.0221 (18)	0.040 (2)	0.0308 (19)	0.0060 (17)	0.0055 (15)	−0.0011 (16)
C20	0.0193 (18)	0.0292 (18)	0.0274 (18)	0.0049 (14)	0.0001 (14)	0.0019 (14)
N1	0.0068 (12)	0.0208 (12)	0.0237 (14)	0.0003 (11)	−0.0009 (10)	0.0003 (11)
Fe1	0.0085 (2)	0.0199 (2)	0.0202 (3)	−0.00065 (19)	−0.00110 (17)	0.00174 (18)

### Geometric parameters (Å, º)

**Table:**

C1—C2	1.378 (4)	C10—Fe1	2.047 (3)
C1—N1	1.384 (3)	C10—H10	0.9300
C1—H1	0.9300	C11—C12	1.409 (5)
C2—C3	1.425 (4)	C11—Fe1	2.036 (3)
C2—C5	1.449 (4)	C11—H11	0.9300
C3—C4	1.349 (4)	C12—C13	1.417 (4)
C3—H3	0.9300	C12—Fe1	2.032 (3)
C4—N1	1.373 (4)	C12—H12	0.9300
C4—H4	0.9300	C13—C14	1.417 (4)
C5—C9	1.435 (4)	C13—Fe1	2.046 (3)
C5—C6	1.435 (4)	C13—H13	0.9300
C5—Fe1	2.075 (3)	C14—Fe1	2.053 (3)
C6—C7	1.414 (4)	C14—H14	0.9300
C6—Fe1	2.046 (3)	C15—C16	1.384 (4)
C6—H6	0.9300	C15—C20	1.392 (4)
C7—C8	1.417 (4)	C15—N1	1.424 (4)
C7—Fe1	2.040 (3)	C16—C17	1.389 (4)
C7—H7	0.9300	C16—H16	0.9300
C8—C9	1.413 (4)	C17—C18	1.379 (4)
C8—Fe1	2.038 (3)	C17—H17	0.9300
C8—H8	0.9300	C18—C19	1.378 (4)
C9—Fe1	2.047 (3)	C18—H18	0.9300
C9—H9	0.9300	C19—C20	1.376 (4)
C10—C14	1.403 (4)	C19—H19	0.9300
C10—C11	1.417 (4)	C20—H20	0.9300
			
C2—C1—N1	108.4 (2)	C10—C14—H14	125.9
C2—C1—H1	125.8	C13—C14—H14	125.9
N1—C1—H1	125.8	Fe1—C14—H14	126.4
C1—C2—C3	106.2 (2)	C16—C15—C20	119.2 (3)
C1—C2—C5	127.7 (3)	C16—C15—N1	120.6 (3)
C3—C2—C5	125.9 (3)	C20—C15—N1	120.2 (3)
C4—C3—C2	108.3 (3)	C15—C16—C17	120.1 (3)
C4—C3—H3	125.9	C15—C16—H16	120.0
C2—C3—H3	125.9	C17—C16—H16	120.0
C3—C4—N1	108.9 (3)	C18—C17—C16	120.7 (3)
C3—C4—H4	125.6	C18—C17—H17	119.6
N1—C4—H4	125.6	C16—C17—H17	119.6
C9—C5—C6	106.4 (3)	C19—C18—C17	118.7 (3)
C9—C5—C2	128.5 (3)	C19—C18—H18	120.6
C6—C5—C2	125.0 (3)	C17—C18—H18	120.6
C9—C5—Fe1	68.60 (16)	C20—C19—C18	121.4 (3)
C6—C5—Fe1	68.56 (16)	C20—C19—H19	119.3
C2—C5—Fe1	130.1 (2)	C18—C19—H19	119.3
C7—C6—C5	108.7 (3)	C19—C20—C15	119.8 (3)
C7—C6—Fe1	69.53 (17)	C19—C20—H20	120.1
C5—C6—Fe1	70.70 (16)	C15—C20—H20	120.1
C7—C6—H6	125.7	C4—N1—C1	108.2 (2)
C5—C6—H6	125.7	C4—N1—C15	126.0 (2)
Fe1—C6—H6	125.7	C1—N1—C15	125.7 (2)
C6—C7—C8	108.1 (3)	C12—Fe1—C11	40.53 (13)
C6—C7—Fe1	69.99 (17)	C12—Fe1—C8	127.87 (13)
C8—C7—Fe1	69.56 (17)	C11—Fe1—C8	165.53 (13)
C6—C7—H7	126.0	C12—Fe1—C7	107.52 (13)
C8—C7—H7	126.0	C11—Fe1—C7	127.38 (13)
Fe1—C7—H7	126.1	C8—Fe1—C7	40.67 (12)
C9—C8—C7	108.2 (3)	C12—Fe1—C13	40.66 (13)
C9—C8—Fe1	70.14 (16)	C11—Fe1—C13	68.05 (13)
C7—C8—Fe1	69.77 (17)	C8—Fe1—C13	108.58 (12)
C9—C8—H8	125.9	C7—Fe1—C13	118.60 (13)
C7—C8—H8	125.9	C12—Fe1—C6	117.74 (13)
Fe1—C8—H8	125.8	C11—Fe1—C6	107.65 (12)
C8—C9—C5	108.6 (3)	C8—Fe1—C6	68.25 (12)
C8—C9—Fe1	69.40 (17)	C7—Fe1—C6	40.48 (11)
C5—C9—Fe1	70.67 (16)	C13—Fe1—C6	151.85 (13)
C8—C9—H9	125.7	C12—Fe1—C10	68.21 (13)
C5—C9—H9	125.7	C11—Fe1—C10	40.61 (13)
Fe1—C9—H9	125.8	C8—Fe1—C10	152.65 (13)
C14—C10—C11	108.1 (3)	C7—Fe1—C10	165.59 (12)
C14—C10—Fe1	70.23 (18)	C13—Fe1—C10	67.81 (13)
C11—C10—Fe1	69.28 (18)	C6—Fe1—C10	128.09 (12)
C14—C10—H10	125.9	C12—Fe1—C9	166.10 (13)
C11—C10—H10	125.9	C11—Fe1—C9	152.46 (13)
Fe1—C10—H10	126.1	C8—Fe1—C9	40.47 (11)
C12—C11—C10	108.1 (3)	C7—Fe1—C9	68.23 (12)
C12—C11—Fe1	69.58 (18)	C13—Fe1—C9	128.57 (12)
C10—C11—Fe1	70.12 (17)	C6—Fe1—C9	68.27 (12)
C12—C11—H11	126.0	C10—Fe1—C9	119.17 (13)
C10—C11—H11	126.0	C12—Fe1—C14	68.19 (13)
Fe1—C11—H11	125.9	C11—Fe1—C14	67.88 (13)
C11—C12—C13	107.9 (3)	C8—Fe1—C14	119.39 (12)
C11—C12—Fe1	69.89 (19)	C7—Fe1—C14	152.72 (13)
C13—C12—Fe1	70.22 (18)	C13—Fe1—C14	40.43 (12)
C11—C12—H12	126.1	C6—Fe1—C14	165.97 (12)
C13—C12—H12	126.1	C10—Fe1—C14	40.03 (12)
Fe1—C12—H12	125.4	C9—Fe1—C14	109.11 (12)
C14—C13—C12	107.8 (3)	C12—Fe1—C5	151.56 (12)
C14—C13—Fe1	70.06 (18)	C11—Fe1—C5	118.19 (12)
C12—C13—Fe1	69.12 (18)	C8—Fe1—C5	68.44 (12)
C14—C13—H13	126.1	C7—Fe1—C5	68.45 (12)
C12—C13—H13	126.1	C13—Fe1—C5	166.39 (12)
Fe1—C13—H13	126.3	C6—Fe1—C5	40.74 (11)
C10—C14—C13	108.1 (3)	C10—Fe1—C5	108.33 (12)
C10—C14—Fe1	69.73 (18)	C9—Fe1—C5	40.73 (11)
C13—C14—Fe1	69.51 (18)	C14—Fe1—C5	128.38 (12)
			
N1—C1—C2—C3	0.9 (3)	Fe1—C10—C11—C12	59.5 (2)
N1—C1—C2—C5	176.7 (3)	C14—C10—C11—Fe1	−59.7 (2)
C1—C2—C3—C4	−0.8 (3)	C10—C11—C12—C13	0.4 (4)
C5—C2—C3—C4	−176.7 (3)	Fe1—C11—C12—C13	60.2 (2)
C2—C3—C4—N1	0.4 (3)	C10—C11—C12—Fe1	−59.8 (2)
C1—C2—C5—C9	−5.1 (5)	C11—C12—C13—C14	−0.4 (4)
C3—C2—C5—C9	169.9 (3)	Fe1—C12—C13—C14	59.6 (2)
C1—C2—C5—C6	178.8 (3)	C11—C12—C13—Fe1	−60.0 (2)
C3—C2—C5—C6	−6.2 (5)	C11—C10—C14—C13	0.0 (3)
C1—C2—C5—Fe1	88.4 (4)	Fe1—C10—C14—C13	−59.1 (2)
C3—C2—C5—Fe1	−96.6 (3)	C11—C10—C14—Fe1	59.1 (2)
C9—C5—C6—C7	−1.0 (3)	C12—C13—C14—C10	0.3 (3)
C2—C5—C6—C7	175.9 (3)	Fe1—C13—C14—C10	59.3 (2)
Fe1—C5—C6—C7	−59.4 (2)	C12—C13—C14—Fe1	−59.0 (2)
C9—C5—C6—Fe1	58.4 (2)	C20—C15—C16—C17	0.6 (5)
C2—C5—C6—Fe1	−124.8 (3)	N1—C15—C16—C17	179.3 (3)
C5—C6—C7—C8	0.7 (3)	C15—C16—C17—C18	−0.6 (5)
Fe1—C6—C7—C8	−59.4 (2)	C16—C17—C18—C19	0.4 (5)
C5—C6—C7—Fe1	60.1 (2)	C17—C18—C19—C20	−0.1 (5)
C6—C7—C8—C9	−0.2 (3)	C18—C19—C20—C15	0.0 (5)
Fe1—C7—C8—C9	−59.8 (2)	C16—C15—C20—C19	−0.3 (5)
C6—C7—C8—Fe1	59.6 (2)	N1—C15—C20—C19	−179.0 (3)
C7—C8—C9—C5	−0.4 (3)	C3—C4—N1—C1	0.2 (3)
Fe1—C8—C9—C5	−60.0 (2)	C3—C4—N1—C15	177.4 (3)
C7—C8—C9—Fe1	59.6 (2)	C2—C1—N1—C4	−0.7 (3)
C6—C5—C9—C8	0.8 (3)	C2—C1—N1—C15	−177.9 (3)
C2—C5—C9—C8	−175.8 (3)	C16—C15—N1—C4	−178.3 (3)
Fe1—C5—C9—C8	59.2 (2)	C20—C15—N1—C4	0.5 (4)
C6—C5—C9—Fe1	−58.38 (19)	C16—C15—N1—C1	−1.6 (4)
C2—C5—C9—Fe1	125.0 (3)	C20—C15—N1—C1	177.2 (3)
C14—C10—C11—C12	−0.3 (4)		
