# Crystal structure of 3-ferrocenyl-1-phenyl-1*H*-pyrrole, [Fe(η^5^-C_5_H_4_^*c*^C_4_H_3_ *N*Ph)(η^5^-C_5_H_5_)]. Corrigendum

**DOI:** 10.1107/S2056989016005624

**Published:** 2016-05-27

**Authors:** Ulrike Pfaff, Marcus Korb, Heinrich Lang

**Affiliations:** a Technische Universität Chemnitz, Fakultät für Naturwissenschaften, Institut für Chemie, Anorganische Chemie, D-09107 Chemnitz, Germany

## Abstract

In the paper by Pfaff *et al.* [*Acta Cryst.* (2016), E**72**, 92–95], the acknowledgements section is incomplete.

## Abstract 2

Erratum to *Acta Cryst.* (2016), E**72**, 92–95.

The acknowledgements section of Pfaff *et al.* (2016) ▸ is incomplete. The full version is as follows:

MK thanks the Fonds der Chemischen Industrie for a PhD Chemiefonds fellowship. The publication costs of this article were funded by the German Research Foundation/DFG and the Technische Universität Chemnitz in the funding program Open Access Publishing.
